# Possible correlated signaling pathways with chronic urate nephropathy: A review

**DOI:** 10.1097/MD.0000000000034540

**Published:** 2023-08-11

**Authors:** Kaiqing Li, Yanchun Ma, Xue Xia, Huili Huang, Jianing Li, Xiaoxin Wang, Yang Gao, Shuxiang Zhang, Tong Fu, Ying Tong

**Affiliations:** a Heilongjiang University of Traditional Chinese Medicine, Harbin, China; b Institute of Traditional Chinese Medicine, Heilongjiang University of Chinese Medicine, Harbin, China; c Brandeis University, Waltham, MA; d First Affiliated Hospital, Heilongjiang University of Chinese Medicine, Harbin, China.

**Keywords:** action mechanism, hyperuricemia nephropathy, signal pathway, uric acid

## Abstract

Hyperuricemia nephropathy, also known as gouty nephropathy, refers to renal damage induced by hyperuricemia caused by excessive production of serum uric acid or low excretion of uric acid. the persistence of symptoms will lead to changes in renal tubular phenotype and accelerate the progress of renal fibrosis. The existence and progressive aggravation of symptoms will bring a heavy burden to patients, their families and society, affect their quality of life and reduce their well-being. With the increase of reports on hyperuricemia nephropathy, the importance of related signal pathways in the pathogenesis of hyperuricemia nephropathy is becoming more and more obvious, but most studies are limited to the upper and lower mediating relationship between 1 or 2 signal pathways. The research on the comprehensiveness of signal pathways and the breadth of crosstalk between signal pathways is limited. By synthesizing the research results of signal pathways related to hyperuricemia nephropathy in recent years, this paper will explore the specific mechanism of hyperuricemia nephropathy, and provide new ideas and methods for the treatment of hyperuricemia nephropathy based on a variety of signal pathway crosstalk and personal prospects.

## 1. Introduction

Uric acid (UA) is a product that cannot be decomposed by human beings. The production of UA is closely related to endogenous purine nucleotide metabolism and exogenous dietary intake. The physiological concentration of soluble UA has cartilage protection and anti-inflammatory effects.^[1]^ The excretion of UA is related to the kidney and intestinal tract, of which about 2/3 of UA is excreted through the kidney and 1/3 of the UA is excreted from the intestine.^[2]^ When purine metabolism disorder occurs in the body and UA production is excessive, or renal tubule damage and intestinal flora imbalance result in reduced UA excretion, UA deposits in the blood resulting in excessive serum UA, and decreased UA metabolism in urine leads to reduced UA excretion resulting in hyperuricemia (HUA).^[3]^ HUA has been recognized as an independent risk factor for kidney disease.^[4]^ When serum UA exceeds its saturation in blood or tissue fluid, sodium urate crystals can be formed locally in the joint.^[5]^ The deposition in the kidney is called hyperuricemia nephropathy (HN).^[6]^ HN often occurs in the middle-aged and elderly, with more males than females, and the risk of chronic kidney disease in males with HUA is 2.89 times higher than that in females with normal UA.^[7]^ The regional difference is significant, which is significantly higher in cities than in rural and coastal areas.^[8]^ The prevalence rate of HUA in Tibetan adults on the Qinghai-Tibet Plateau is 40.7%.^[9]^ There is a U-shaped correlation between mortality and HUA levels in China.^[10]^ There is also a U-shaped association between serum UA and all-cause and specific cause mortality in American adults.^[11]^ With the rapid development of modern society and the significant improvement of residents living standards, excessive nutrition intake will increase the burden of metabolic pathways in the body, and the incidence of HN will also increase year by year, which will gradually become a major hidden danger affecting people’s quality of life and threatening human health. Patients with high UA do not take medicine in accordance with the regulations, take it privately, take it indiscriminately, and take it doubly without effect, which will bring burden to the kidney. Induce drug-induced renal damage and increase the risk of metabolic disorders with metabolic syndrome. Therefore, understanding why HN is mediated is very important for the treatment of this disease. This article will explain the possible mechanism of HN in the direction of different HN signal pathways, and summarize it.

## 2. Inflammatory body signal pathway influenced by NLR family

NLR family receptors can be recognized and activated by danger signals, and the NLR family plays an important role in ligand recognition, signal transduction, and the body’s immune response as a class of receptors for intrinsic immunity against pathogenic organisms. Mitochondrial autophagy is a self-protective mechanism that selectively clears damaged mitochondria to maintain homeostasis, and is the main cause of renal tubular injury in gouty nephropathy. Mitochondrial biosynthesis and mitochondrial autophagy flow after injury are the key links in the repair of renal tubular injury. NLRX1 is a member of the NLR family, which plays an important role in regulating immune response, promoting ROS production, mitochondrial damage, autophagy,^[12]^ apoptosis^[13]^ and so on. Because the N-terminal of NLRX1 contains a mitochondrial localization sequence, it becomes the first NLR located in mitochondria and plays an important role in mitochondrial directional transmission.^[14]^NLRX1 can enter the mitochondrial matrix from the outer membrane of mitochondria by means of its N-terminal MT sequence.^[15]^ By interacting with UQCRC2 in respiratory chain complex Ⅲ, ROS can be produced in mitochondria.^[16]^ Excessive ROS will continuously release its own high cytotoxicity. At this time, the body will initiate a series of mechanisms to clear excess ROS, activate autophagy pathway, inhibit inflammation, and maintain the level of homeostasis.

NOD-like receptor domain-related protein 3 (NLRP3) is an important member of the NLR family, which forms inflammatory body complexes with apoptosis-related spot-like proteins and caspase-1 precursor. When NLRP3 senses the active danger signal of activators (UA, blood glucose, potassium, chloride, lysosome damage, mitochondria, endoplasmic reticulum, Golgi apparatus),^[17]^ it binds with proteins in apoptosis-related spot-like proteins to recruit Pro-caspase-1 and then aggregates NLRP3 inflammatory bodies, cleaves Pro-caspase-1 to transform it into active caspase-1, and promotes the maturation of pro-inflammatory cytokines IL-1 β and IL-18. Induce inflammation and promote apoptosis.^[18]^ Cui^[19]^ adopted phloretin to improve renal injury induced by high UA by inhibiting NLRP3 signal pathway and UA reabsorption. Through the activation of HK-2 cells induced by NLRP3 activator, it was found that phloretin could reduce the levels of NLRP3, caspase-1, IL-1 β and IL-18, play the role of anti-inflammation and renal protection. Liu^[20]^ took methyl gallate to inhibit the assembly of NLRP3 inflammatory bodies by blocking the upstream pathway of inflammatory bodies (ROS production), significantly reversed the upregulation of inflammatory body proteins in HN mice, and decreased the expression of TNF- α protein in mice by inhibiting the activation of nuclear factor-kB (NF-KB). Anti-inflammatory effects can also be achieved by inhibiting the pre-activation of NLRP3 inflammatory bodies in humans. Zhang^[21]^ urolithione A interferes with STING-NLRP3 axis-mediated HN to achieve anti-inflammation and promote mitochondrial autophagy to further inhibit the activation of STING-NLRP3 axis. The expression of IL-1 β, IL-6 and TNF- α protein decreased in renal tissue and serum. Through cell culture, it was found that urolithione A could eliminate the inflammation-mediated by STING-NLRP3 axis by re-triggering mitochondrial autophagy pathway. Shui^[22]^ found that the traditional Chinese medicine formula Simiao Pill Module in the treatment of HN can delay the progression of chronic HN by inhibiting the activation of NLRP3 inflammatory bodies and reducing the secretion of inflammatory cytokines by potassium oxalate. Yang^[23]^ found that Wuling Powder could inhibit the activation of NLRP3 and the expression of IL-1 β protein in kidney. Hu^[24]^ blocked autophagy by inhibiting the release of NLRP3 inflammatory bodies and IL-1b and IL-18, and controlled the activation and scorching death of NLRP3 inflammatory bodies by inhibiting autophagy.

NLRP6 is a new member of the NLR family that forms inflammatory bodies, similar to NLPR3, which has 32% similarity in human amino acid sequence and 33%^[25]^ in mice, which can form inflammatory bodies, promote IL-1 β, IL-18 maturation and induce scorch death to play a pro-inflammatory form. NLRP6 is highly expressed in the intestine and plays a role in regulating intestinal homeostasis and anti-inflammation by activating caspases-1, caspases-11 or regulating key transcription factors NF-kB.^[26]^Shen^[27]^ found that double-stranded ribonucleic acid can promote the formation of liquid-liquid phase separation (liquid-liquid phase separation, LLPS) of NLRP6, and LLPS of NLRP6 can promote the activation of inflammatory bodies in intestine and liver. Ji^[28]^ found that NLRP6 controls inflammation by inhibiting macrophage recruitment and NF-kB pathway in the liver, prevents fat accumulation and hepatocyte injury, and acts on intestinal epithelium at the same time to protect the integrity of intestinal epithelium. Lu^[29]^ found that NLRP6 can inhibit inflammation by inhibiting NF- κ B and ERK signal pathways, and negatively regulate the expression of IL-6 and TNF- α proteins in inflammation.

**Figure 1.**
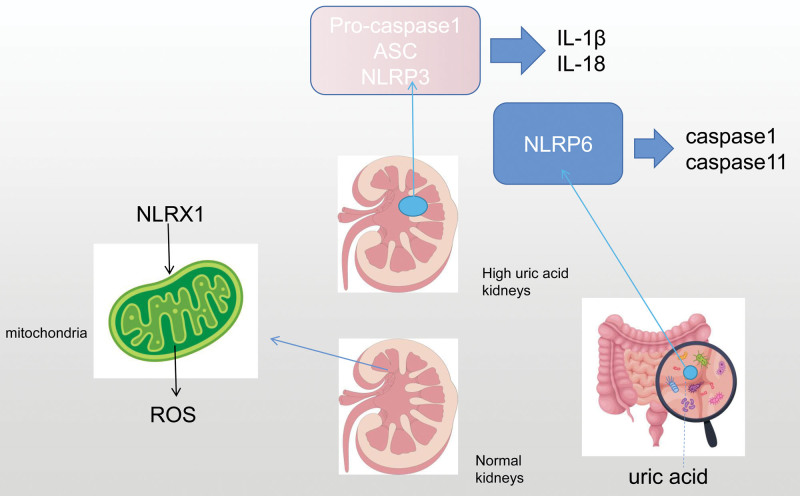
Inflammatory body signal pathway influenced by NLR family.

To sum up, NLRX1, NLRP3 and NLRP6 in the NLR family can regulate the progression of HN by inhibiting inflammatory response and play an important role in inflammatory signaling pathways. See Figure 1 for details.

## 3. Extracellular regulated protein kinase signal pathway

Extracellular regulated protein kinases (ERK1/2) are a class of signal transduction proteins with extensive catalytic activity, which can promote serine/threonine phosphorylation. Phosphorylated ERK1/2 has activity. Affected by upstream protein signals, signals can be transferred from the cytoplasm to the nucleus, such as RAS,^[30]^ RAF,^[31]^ MEK.^[32]^Furthermore, it mediates the transcriptional activation of downstream proteins and participates in cell proliferation and differentiation, senescence and apoptosis,^[33]^ such as Elk-1,^[34]^ ATF^[35]^, and Ap-1.^[36]^ Liu^[37]^ observed the changes of glomeruli and renal interstitium by increasing ERK1/2 phosphorylation in the HN rat model, and found that the pharmacological targeting of ERK2/1 can alleviate HN by inhibiting TGF-βsignal transduction, reducing inflammatory response and inhibiting molecular processes associated with elevated serum UA levels in the body. Tao^[38]^ blocking ERK1/2 signaling pathway through U0126, a selective inhibitor of ERK1/2, can reduce epithelial-mesenchymal transformation and renal tubular cell injury in HN rats, and then induce renal protection, and reduce renal tubular injury by enhancing the resistance of cells to oxidative stress. Xiong^[39]^ adopted bromine domain and terminal extracellular protein small molecule inhibitor to inhibit renal epithelial-mesenchymal transition and inflammation by blocking TGF-β, ERK1/2 and NF-κB signal transduction, which can effectively inhibit ERK1/2 phosphorylation, improve renal function and reduce renal interstitial fibrosis and histological injury in HN rats. Tang^[40]^ inhibited renal interstitial fibrosis by targeting mouse ERK1/2 signal pathway through silent miR-21, and increased the expression of TGF-β1 and p-ERK1/2 by regulating ERK1/2 signal pathway, thus inducing the production of extracellular matrix to slow down renal interstitial fibrosis. Zhou^[41]^ found that astragaloside can alleviate acute renal injury by inhibiting ERK signal pathway in vitro, which is essentially blocking the phosphorylation of ERK to promote renal cell survival. Zha^[42]^ found that UA-induced mesenchymal transformation in renal tubular epithelial cells is caused by NADPH oxidase-mediated ERK1/2 activation and endothelial vasoconstrictor peptide expression, so it improves epithelial-mesenchymal transformation in renal tubular cells by inhibiting NADPH oxidase-ERK1/2- endothelial vasoconstrictor peptide pathway. Shi^[43]^ found that blocking the activation of TGF-β1/Smad3 and epidermal growth factor receptor/ERK1/2 signal pathway can reduce renal fibrosis and apoptosis of renal tubular cells. BRYANT^[44]^ found that inhibition of ERK can damage mitochondrial activity, block mitochondrial fission, increase mitochondrial fusion, increase autophagy flux and autophagy signal, nucleotide metabolism and gene transcription, decrease the quality of intracellular mitochondria, increase mitochondrial pathological flux, and finally activate autophagy to clear damaged mitochondria and inhibit the activation of inflammatory bodies.

**Figure 2.**
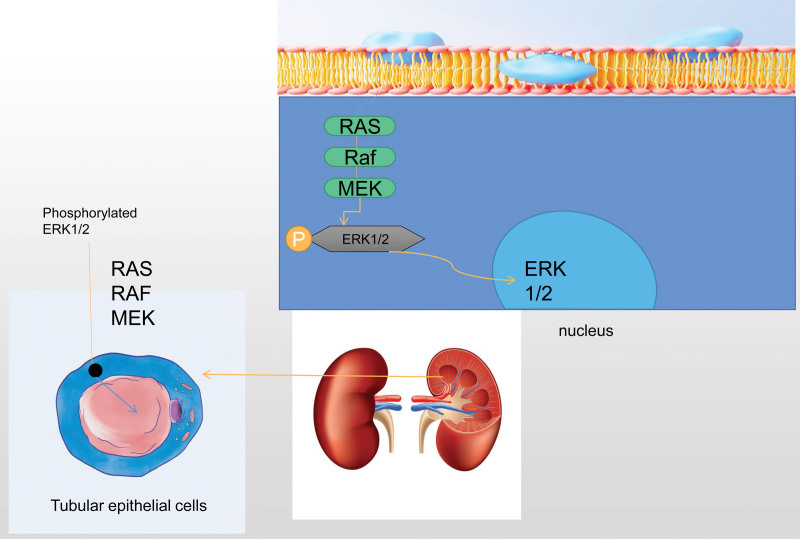
Extracellular regulated protein kinase signal pathway.

To sum up, ERK1/2 signal pathway plays an important role in the development of HN. Some clinical scholars block this signal pathway to inhibit the phosphorylation of ERK1/2 and control its activity, which is helpful to reduce the erosion and damage of renal tubular epithelial cells caused by hyperuric acid, and play an anti-inflammatory and renal protective role. See Figure 2 for details.

## 4. Nuclear factor-KB signal pathway

NF-kB signaling pathway is closely related to the development and prognosis of HN.^[45]^ Activation of NF- kappa B pathway can induce changes in signal transduction pathway,^[46]^ play a role in transcriptional regulation of cytokines,^[47]^ and participate in immune regulation,^[48]^ inflammation mediation^[49]^ and apoptosis.^[50]^ NF-kB exists widely in the cytoplasm of the body. However, when the NF-kB signal pathway is activated by pro-inflammatory signals and specific receptors by multi-loop target control, NF-kB will transfer from the cytoplasm to the nucleus and initiate gene regulation and transcription.^[51]^ Among them, pro-inflammatory signals include ROS,^[52]^ UA,^[53]^ TNFα,^[54]^ LPS,^[55]^ and IL-1β.^[56]^ By binding to cell surface receptors, they recruit downstream proteins and promote self-ubiquitination, induce IKK complex activation, promote IKBa phosphorylation to degrade proteases, and form NF-kB to participate in the nuclear transcription and regulation of cytokines. Specific receptors include LTβR,^[57]^ CD40,^[58]^ CD27,^[59]^ CD30,^[60]^ BAFF,^[61]^ RANK.^[62]^ By recruiting NF- kappa B to induce kinase to phosphorylate and activate IKKαcomplex,^[63]^ further induce phosphorylation of p52 precursor and P100, promote protein maturation and RelB:p52 transfer to nuclear activation target gene.^[64]^ Saral^[65]^ found that white tea infusion can prevent cisplatin induced renal damage by inhibiting the level of NF-kB and reducing the expression of TNF-αand IL-6 protein. Lu^[66]^ found that galangin could control UA-induced renal tubular epithelial cell damage and renal inflammation by inhibiting the activation of NF-kB signal pathway. Liu^[67]^ found that UA induces mesenchymal transformation of renal tubular epithelial cells by activating NF-kB signal pathway, and up-regulates the levels of IL-6,IL-1βand TNF-αproteins. Renal interstitial fibrosis and epithelial-interstitial transition can be reversed by inhibiting the activation of NF-kB signal pathway. Liu^[68]^ found that CRY1 is the target gene of miR-181a. Upregulation of miR-181a can down-regulate the expression of CRY1 protein and promote the excretion of CRY1 by inhibiting NF-kB signal pathway, thus alleviating glomerulosclerosis and tubular epithelial injury in rats with chronic kidney disease. Wan^[69]^ protect the kidney by inhibiting the activation of TLR4/MyD88/NF-kB signal pathway to promote UA excretion and control the biosynthesis of UA and the expression of intercellular inflammatory factors. Chen^[70]^ improve renal inflammation and reduce excessive renal damage caused by the secretion of inflammatory cytokines by inhibiting LPS/TLR4/NF-kB inflammatory signal pathway induced by lipid emulsion in HUA rats. Hassan^[71]^ found that renal tubulointerstitial fibrosis induced by unilateral ureteral obstruction could be alleviated by inhibiting STAT3/NF-kB signal transduction. Wang^[72]^ found that rosiglitazone can reduce inflammation and inhibit the transformation of epithelium to stroma in HN rat model by inhibiting the activation of TGF-βand NF-kB signal pathways, thus slowing down the progression of HN. Shi^[73]^ take fatty acid binding protein 4 inhibitors to slow down the degree of renal inflammation and fibrosis by regulating the activation of JAK2-STAT3-NF-kB-P65 signaling pathway. Pan^[74]^ found that inhibiting the activation of NF-kB, ASK1/JNK/c-Jun, and JAK2/STAT3 signal pathways can reduce the release of TNF-αin the kidney of HN mice and reduce renal fibrosis and inflammatory infiltration.

**Figure 3.**
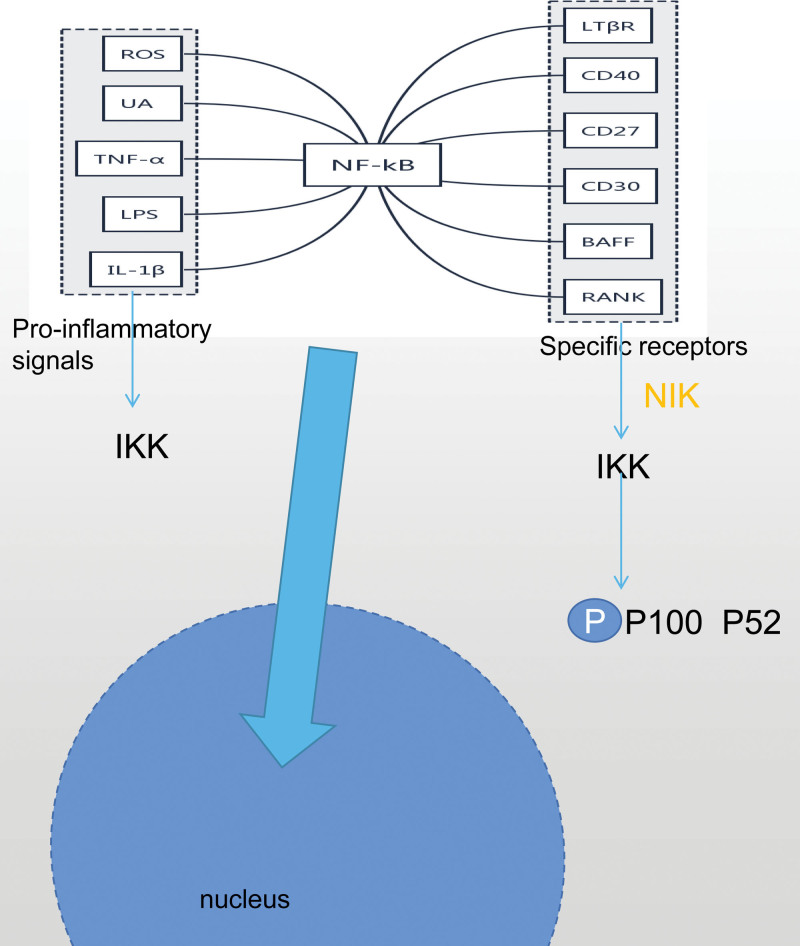
Nuclear factor-KB signal pathway.

To sum up, inhibiting the activation of NF-kB signal pathway can slow down the progress of HN, which is embodied in the synergism between upstream and downstream of NF-kB signal pathway, which can inhibit renal fibrosis by interfering with excessive transcription from cytoplasm to nucleus, control inflammatory response, regulate immune factors and activate immune response, promote UA excretion and protect kidney. See Figure 3 for details.

## 5. Toll-like receptor signaling pathway

Toll-like receptors (TLR) can mediate immune response by recognizing different related molecular patterns, participate in inflammatory response^[75]^ and immune cell regulation,^[76]^ and are closely related to innate immune response^[77]^ and adaptive immune response.^[78]^ When the TLR signaling pathway is stimulated by UA,^[79]^ LPS,^[80]^ and peptidoglycan,^[81]^ the TLR can protect the host by recognizing pathogens and activating downstream pathways to mediate immune responses. TLR signaling pathway is associated with TRIF,^[82]^ TRAF3^[83]^ class MyD88 nondependent pathway and MyD88^[84]^ induced activation of P38,^[85]^ JNK,^[86]^ Mitogen-activated protein kinase (MAPK),^[87]^ TAK1/TBK1,^[88]^ NF-kB,^[89]^ and IRF5^[90]^ activation are closely related. The persistence of high UA state will cause phenotypic changes of renal tubular epithelial cells and renal fibrosis.^[91]^ As an independent risk factor affecting renal function, UA participates in the whole process of damage and can be recognized by TLR in the innate immune system, and then activate TLR signal pathway to mediate immune response and the expression of inflammatory factors. Correa-Costa^[92]^ found that inhibition of TLR-MyD88- inflammatory corpuscle complex signal pathway can delay the progression of tubulointerstitial glomerulonephritis and play a role in renal protection and anti-inflammation. Wu^[93]^ take rhubarb to regulate immune inflammation and renal protection in HN rats by inhibiting the activation of key factors of TLR/MyD88/NF-KB signal pathway. Liu^[67]^ found that UA can promote the production of inflammatory cytokines and induce EMT in renal tubular epithelial cells by activating TLR4/NF-kB signal pathway. Targeted intervention of the activation of TLR4/NF-kB signal pathway can effectively inhibit the occurrence of EMT in HN and the progress of renal interstitial fibrosis. Liu^[68]^ found that miR-181a can inhibit the activation of TLR/NF-KB signal pathway by down-regulating the level of target gene CRY1, delay the progression of glomerulosclerosis and tubular epithelial injury in chronic kidney disease, reduce the degree of renal injury and promote renal homeostasis. Ma^[94]^ found that Polygonum cuspidatum can improve UA-induced renal injury by inhibiting the biological activities of TLR4, NLRP3 and MCP-1. Milanesi^[95]^ found that angiotensin II can induce renal tubular injury through interaction with TLR4, and the inhibition of TLR4 can reduce inflammation and oxidative stress induced by UA and angiotensin II. Madbouly^[79]^ found that taurine reduced the deposition of extracellular matrix and improved renal fibrosis by inhibiting the expression of TLR4 in renal tubular epithelial cells.

**Figure 4.**
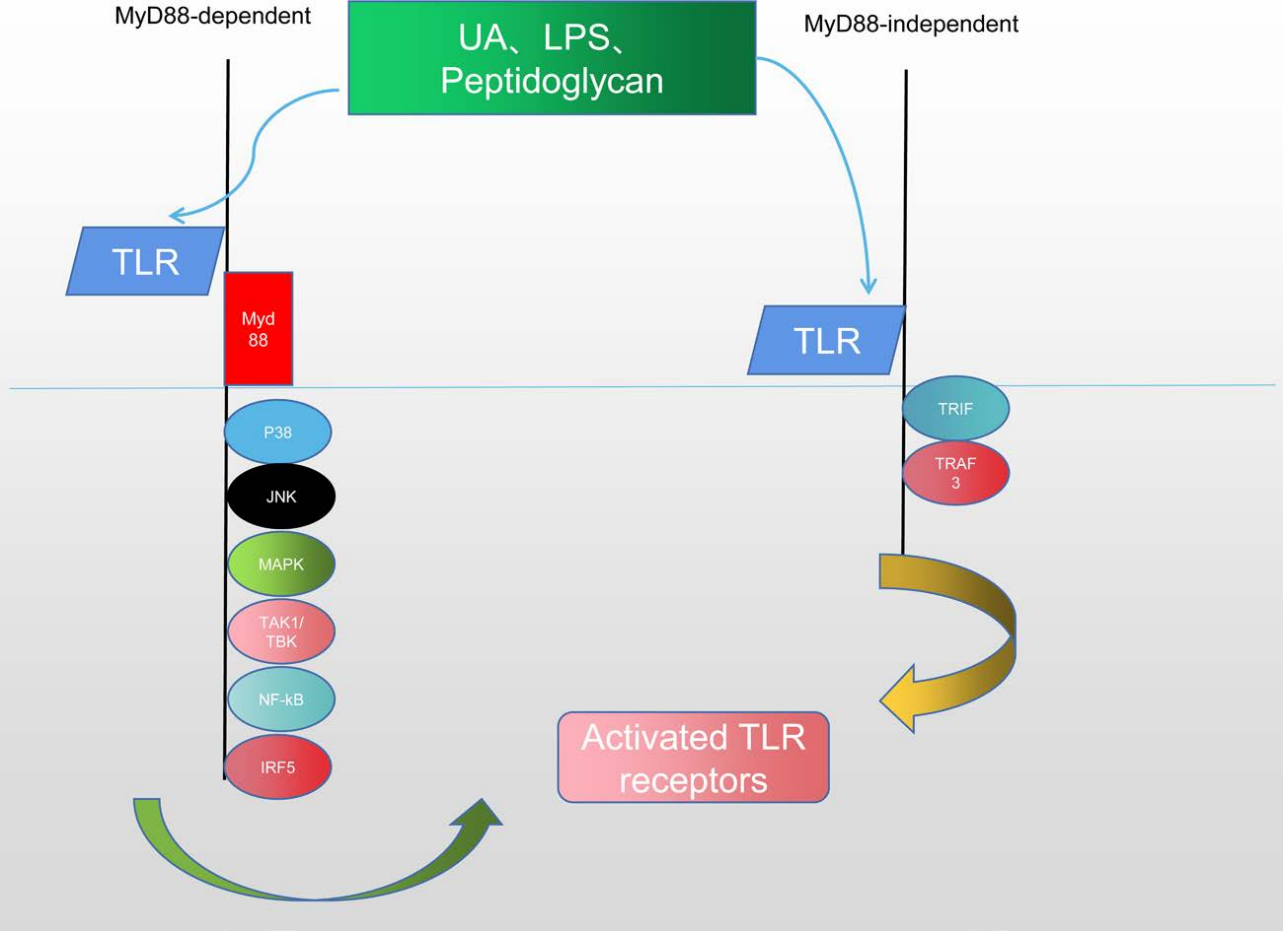
TLR receptors are associated with MyD88-dependent/independent signal transduction pathways. TLR = toll-like receptors.

To sum up, UA can improve the progress of HN and injury of renal tubular epithelial cells by activating Toll-like receptor signal pathway and activating immune response through downstream pathway, which may provide new targets and ideas for drug therapy of HN. See Figure 4 for details.

## 6. Mitogen-activated protein kinase signal pathway

MAPK plays an important role in signal transduction. MAPK can be stimulated by a variety of extracellular stimuli (growth factors,^[96]^ cytokines,^[97]^ lipotoxicity,^[98]^ rate threshold^[99]^) and activate serine-threonine protein kinases to transduce cell surface signals into the nucleus.^[100]^ The key to the intracellular phosphorylation cascade is the activation of the MAPK signaling pathway, which receives extracellular signals to act on membrane receptors and activates downstream target gene transcription factors (ERK,^[101]^ p38,^[102]^ JNK,^[103]^ ERK5^[104]^) through protein factors (RAS,^[105]^ MAP3K,^[106]^ MAP2K,^[107]^ MAPK^[108]^) which in turn regulate cell proliferation,^[109]^ differentiation,^[110]^ inflammation-mediated^[111]^ and apoptosis.^[112]^ The pathological basis of HN is that excessive deposition of urate induces phenotypic changes in renal tubular epithelial cell injury. Luo^[113]^ found that the deposition of UA single crystal can induce the activation of MAPK. Wu^[114]^ found that HUA can increase renal autophagy and activate MAPK/ERK signal pathway to inhibit autophagy. In HUA rats, it was found that the phosphorylation level of JNK and ERK1/2 in MAPK signal pathway increased, and autophagy inhibitors down-regulated ERK and JNK signal pathway to improve HUA renal insufficiency and alleviate renal histopathological changes. Yang^[115]^ found that inhibition of IL-36αactivation can block the phosphorylation of P38MAPK, JNK2/1, and ERK1/2 in macrophages, thus reducing renal tubulointerstitial lesions. Xu^[116]^ found that Cortex Phellodendri significantly inhibited the protein levels of MAPK8, MAPK3, and c-Jun in HUA mouse model, and the results were consistent with the results of network pharmacological analysis. UA and other substances can be recognized by TLR and activate MAPK-NLRP3-NF-KB signal pathway, thus play the role of anti-inflammation, inhibition of apoptosis and protection of kidney. Zhang^[117]^ found that ethyl acetate extract of Salvia miltiorrhiza and tanshinone IIA can alleviate renal injury in HN by down-regulating ROS-activated MAPK signal pathway both in vivo and in vitro. Li^[118]^ took salidroside to inhibit the inflammatory reaction and EMT of HK-4 cells induced by TGF-β2 by blocking MAPK signal pathway, and improve the injury of renal tubules and the deposition of extracellular matrix. Sal significantly inhibited the expression of TLR4, p38, ERK and JNK in the mouse model and improved renal fibrosis.

**Figure 5.**
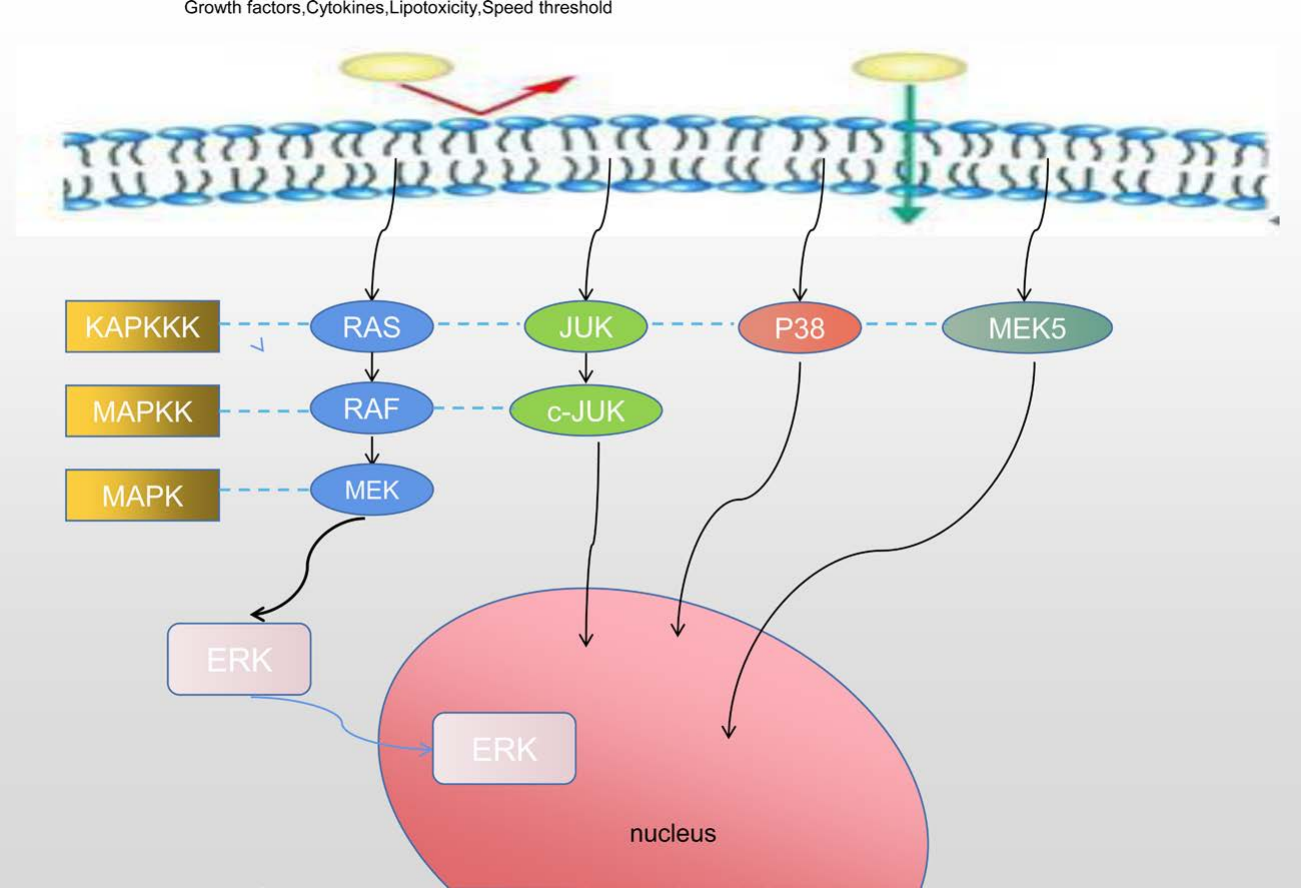
Mitogen-activated protein kinase signal pathway.

To sum up, UA extracellular stimulation can induce downstream transcription factors to mediate renal damage by activating MAPK signal pathway, so it can slow down the progress of HN by inhibiting MAPK signal pathway, and it can also be used as a breakthrough in the future to block the upstream signal of MAPK signal pathway and the connection between MAPK and downstream transcription factors to inhibit renal damage caused by HN. See Figure 5 for details.

## 7. Other signal pathways

In addition to the above 5 classical signal pathways, PhoA/ROCK and PI3K/Akt signal pathways also play an important role in the pathogenesis and progression of HN. PhoA/ROCK accelerates the progression of renal injury by promoting the proliferation and migration of glomerular endothelial cells, apoptosis of renal tubular epithelial cells and interstitial fibrosis, activating inflammatory response and oxidative stress.^[119]^ Chen^[120]^ found that activation of RhoA/ROCK signal pathway can induce glomerular endothelial hyperpermeability and renal barrier dysfunction, and the high permeability of glomerular endothelial barrier function can be improved by inhibiting RhoA/ROCK signal pathway. Chen^[121]^ found that YAP is located in the downstream of Rho/ROCK signal pathway, and YAP is closely related to renal tubulointerstitial fibrosis. Renal fibrosis in proximal tubules can be reduced by inhibiting Rho/ROCK signal pathway. The application of P13k/AKT signaling pathway in HN is to protect renal function by regulating biological processes such as apoptosis, inflammation and oxidative stress. Liu^[122]^ found that berberine can reverse UA-induced glomerulonephritis, oxidative stress and mitochondrial apoptosis by inhibiting PI3K/Akt signal pathway, and then improve the progress of HN. Tu^[123]^ found that quercetin alleviates chronic renal failure and minimizes renal fibrosis and apoptosis by targeting PI3k/Akt signaling pathway.

To sum up, P13k/AKT and PhoA/ROCK signaling pathway has important application value in the treatment of HN. It can be used as a new treatment strategy to protect renal function, reduce renal damage caused by UA, and may become a new target for the treatment of HN.

## 8. Discussion

HN is a disease of chronic renal damage induced by HUA due to abnormal UA metabolism.^[124]^ Its main feature is that UA is deposited in the kidney tissue, resulting in glomerular and renal tubule damage, leading to renal insufficiency.^[125]^ The pathogenesis of HN involves multiple signal pathways, including NLR inflammatory body signal pathway, ERK1/2 signal pathway, NF-κB signal pathway, TLR signal pathway, MAPK signal pathway, PhoA/ROCK, and PI3K/Akt signal pathway. The activation of these signal pathways will induce downstream signal factors to cause damage to the kidney. If the HN signal pathway is not blocked-in time, it is easy to aggravate HN and progress to renal failure. Clinical intervention can be carried out through crosstalk between multiple signal pathways to understand the interaction and provide basic inspiration and innovative ideas for researchers, and to develop diagnostic and therapeutic drugs that can act on multiple signal pathways at the same time according to the differences of crosstalk targets between pathways, so as to provide a new scheme for the treatment of HN.

Although the current research on HN is relatively extensive, and the pathogenetic signal pathway involved is also more specific, the author believes that the intervention and treatment of HN can also be carried out through the following ways. For example, by regulating the relationship between intestinal flora and mitochondrial homeostasis, reverse the progress of HN. The imbalance of mitochondrial homeostasis is the main cause of renal tubular damage in HN,^[126]^ and the intestinal flora is closely related to the inflammatory damage of HN.^[127]^ Mitochondrial homeostasis is the basis for maintaining normal physiological function of cells.^[128]^ When stimulated by danger signals, mitochondria can activate mitochondria with abnormal autophagy clearance function and maintain cell homeostasis.^[129]^ Mitochondrial homeostasis plays a decisive role in the regulation of cell energy metabolism and signal pathway.^[130]^ Among them, mitochondrial biosynthesis and mitochondrial autophagy after injury are the key links in the repair of renal tubular injury. UA-induced phenotypic changes of renal tubular epithelial cells play an important role in the pathogenesis of HN.^[67]^ Mitochondrial homeostasis disorder and activation of autophagy is an important therapeutic target for the prevention and treatment of inflammatory damage of HN.^[131]^ Mitochondria and intestinal microorganisms have homologous characteristics, bacteria and mitochondrial protein targeting sequences show similarity and similar pedigree, resulting in mitochondria becoming the target of intestinal microflora.^[132]^ Therefore, on the basis of the above, this paper puts forward the following hypotheses: excessive deposition of UA, metabolic disorder of intestinal flora, decreased abundance of related bacteria, imbalance of mitochondrial homeostasis, abnormal activation of autophagy, progressive aggravation of renal tubular inflammatory injury, and the pathological process of HN. Therefore, the author puts forward the following thinking: from the point of view of the interaction between metabolites of intestinal flora and mitochondrial homeostasis, by increasing the bacterial abundance directly or indirectly related to HN in intestinal flora, we can improve intestinal flora to maintain mitochondrial homeostasis, inhibit mitochondrial autophagy, protect the normal operation of renal tubule function, and slow down the progress of HN. This may provide a new idea for the treatment of HN.

## Author contributions

**Conceptualization:** Yanchun Ma.

**Formal analysis:** Jianing Li, Shuxiang Zhang.

**Investigation:** Huili Huang.

**Methodology:** Xue Xia.

**Resources:** Xiaoxin Wang.

**Validation:** Yang Gao.

**Visualization:** Tong Fu.

**Writing – original draft:** Kaiqing Li.

**Writing – review & editing:** Ying Tong.
